# Life-Threatening Pulmonary Hemorrhage Responds to Recombinant Factor VIIa: A Case Series in South Florida Hospitals

**DOI:** 10.7759/cureus.6202

**Published:** 2019-11-19

**Authors:** Raiko Diaz, Patricia Almeida, Michael Alvarez, Gustavo Ferrer, Felix Hernandez

**Affiliations:** 1 Pulmonary Medicine, Aventura Hospital and Medical Center, Aventura, USA; 2 Pulmonary and Critical Care, Tampa General Hospital, Tampa, USA; 3 Pulmonary and Critical Care, Aventura Hospital and Medical Center, Aventura, USA

**Keywords:** critical care, diffuse alveolar hemorrhage, recombinant factor vii

## Abstract

Intravenous recombinant activated Factor VIIa (rFVIIa) is approved as a hemostatic agent for only a few bleeding disorders. Since the first reported case of off-label use for rFVIIa in 1999, off-label use far exceeds the use for approved conditions. The endobronchial administration of rFVIIa to control alveolar hemorrhage has been published in only a few case reports. Herein we report a case series of endobronchial rFVIIa use for life-threatening pulmonary hemorrhage at two institutions in south Florida.

## Introduction

Recombinant activated human factor VII (rFVIIa), commercially known as NovoSeven®, is produced by the transfection of the human factor VII gene into clustered hamster cells. The United States Food and Drug Administration (FDA) has currently approved the use of this hemostatic agent for bleeding associated with hemophilia with antibody inhibitors to factors VIII or IX, acquired hemophilia, and congenital factor VII deficiency. In Europe, an additional indication is for the treatment of Glanzmann’s thrombasthenia [1]. Recombinant activated human factor VII is known to exert its effect on the extrinsic pathway of the coagulation cascade by promoting the tissue factor-mediated activation of factor X to Xa. However, at supra-physiologic doses, rFVIIa binds to the surfaces of activated platelets and hastens the conversion of factor Xa to thrombin, thus facilitating hemostasis [2]. Since the first reported case of successful use of intravenous recombinant active human factor VII to stop bleeding in a patient hemorrhaging from gunshot wound, the off-label use of rFVIIa has become increasingly more widespread [3]. Moreover, local applications of rFVIIa to endobronchial mucosa has resulted in bleeding cessation in cases of diffuse alveolar hemorrhage (DAH). The off-label use of rFVIIa serves as a beneficial hemostatic agent in the setting of life-threatening bleeds. However, systemic use poses an additional risk of thromboembolic events. It is hypothesized that the localized use of rFVIIa, as with the management of endobronchial bleeding, will still potentiate its hemostatic effects with less thrombotic risks [4-6]. We describe four cases of diffuse alveolar hemorrhage refractory to conventional therapy where endobronchial rFVIIa was successfully utilized to halt massive bleeding. 

## Case presentation

Case 1

**Figure 1:**
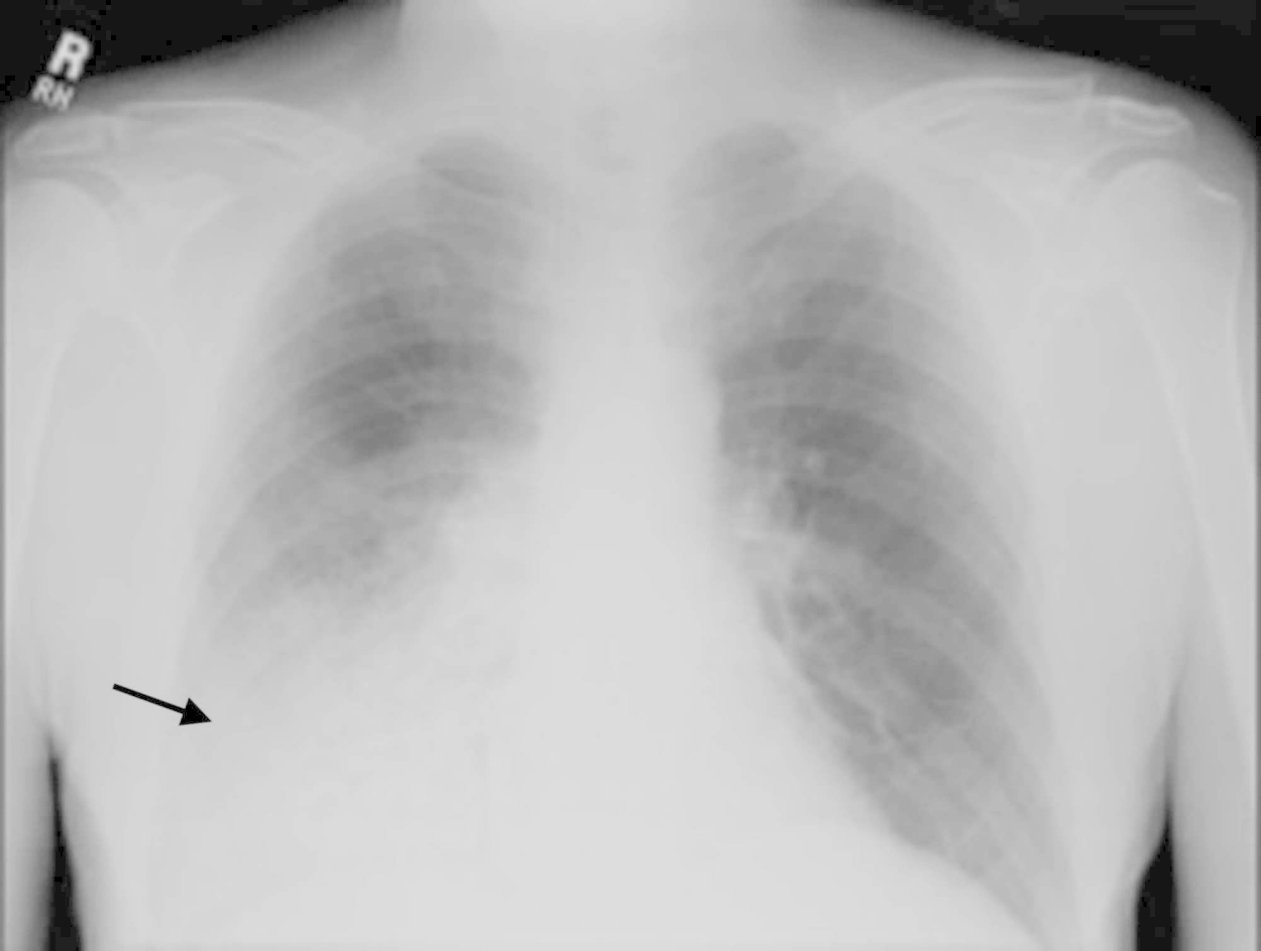
Posteroanterior radiograph of the chest shows right lower lung air-space consolidation. There is no significant enlargement of the cardiac silhouette, nor pleural effusions associated.

**Figure 2:**
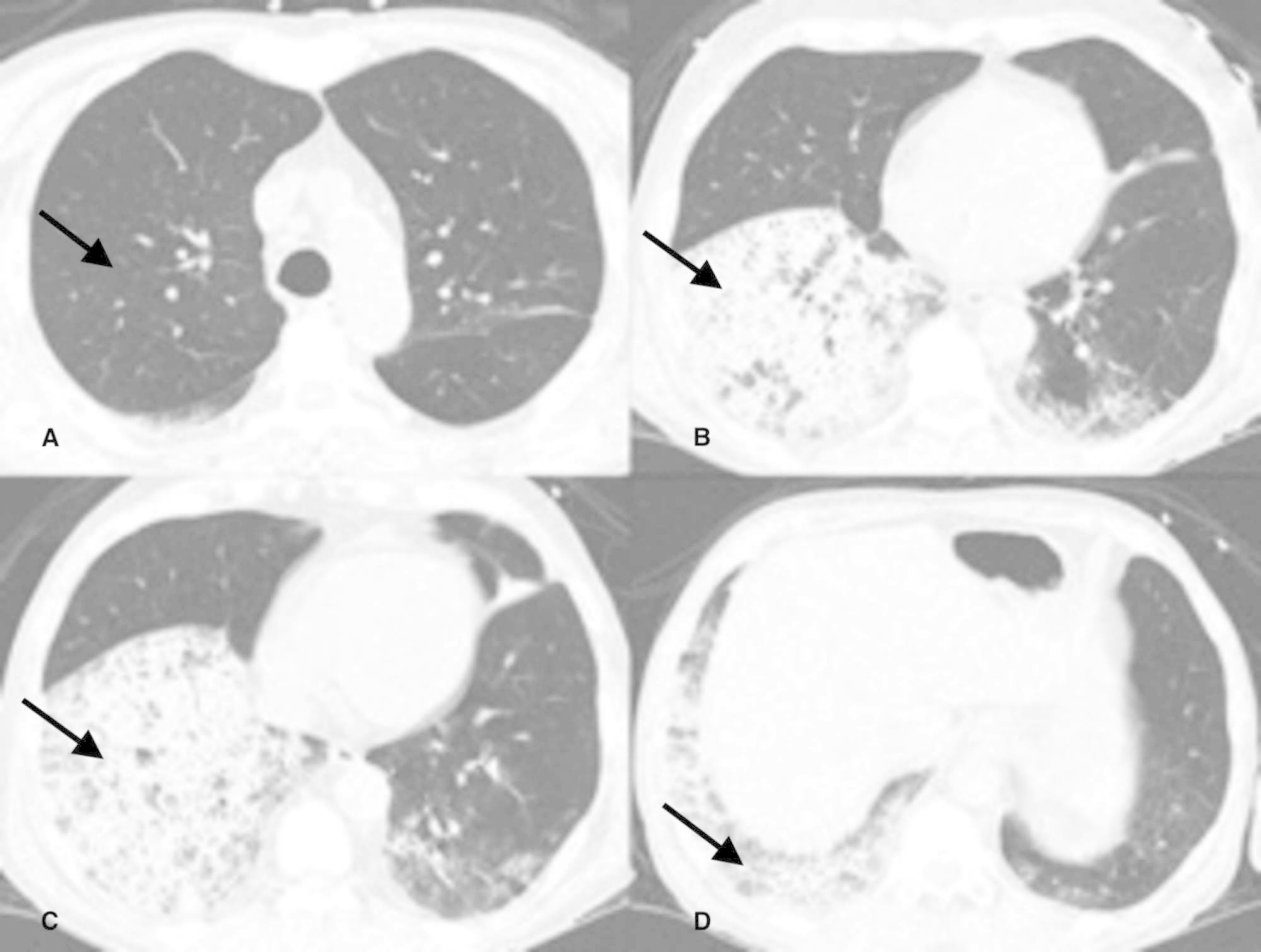
Chest CT scan with 1mm collimation axial images at multiple levels of the thorax in lung window. Image A. Right upper and lower lobes are free of parenchymal disease. Images B, C, D show confluent opacities in the right lower lobe. Black arrows indicate the disease process.

A 67-year-old Jehovah's Witness man with a twenty-six-year history of granulomatosis with polyangiitis (GPA) in remission was admitted to the intensive care unit (ICU) with dyspnea, hemoptysis, acute renal failure, and a hemoglobin level of 5.0 g/dL. He had been treated for ten years with high doses of prednisone and cyclophosphamide until the acute episode. Chest radiograph (Figure 1) and computed tomography (CT) of the chest (Figures 2A-2D) showed complete opacification of the right lower lobe.

A bronchoscopy done at another institution one month prior revealed endobronchial GPA, with narrowing of the left upper lobe bronchus. No bleeding was noted at that time. The patient underwent a repeat bronchoscopy at our institution to exclude an infectious process in preparation for pulse steroids and cyclophosphamide. The findings of active endobronchial GPA were confirmed. In addition, simultaneous active bleeding was noted from the right lower lobe bronchus. A dose of intravenous desmopressin was given, and pulse steroids with one gram of intravenous (IV) methylprednisolone per day were started. 

The hemoptysis persisted twenty-four hours after treatment. The patient’s respiratory status deteriorated, and endotracheal intubation was required. Bronchoscopy confirmed worsening right lower lobe bleeding. It was decided to use recombinant factor VIIa endobronchially, directed to the right lower lobe sub-segments. A dose of 30 mcg/kg was diluted in 120 ml of sterile saline, and the solution was instilled into each right lower lobe segment, with an almost instantaneous cessation of bleeding. The clinical course continued with the administration of pulse steroids, but additional immunosuppressant agents were not added due to the growth of Enterobacter spp. and methicillin-resistant Staphylococcus aureus from the bronchoalveolar lavage. Instead, piperacillin/tazobactam and vancomycin were started. The patient remained stable, with no further evidence of bleeding and was extubated three days later. His hemoglobin slowly improved, and rituximab was started for maintenance therapy. The patient was uneventfully discharged from the ICU. 

Case 2

A previously healthy 46-year-old woman from New York was airlifted to our facility from Jamaica for further management of respiratory failure and shock due to an unclear etiology. She was visiting relatives in Jamaica and developed an episode of acute febrile illness with diarrhea. Symptoms progressed to multi-organ failure, including respiratory failure requiring intubation and mechanical ventilation. On arrival, she was in a septic shock state requiring multiple vasopressors for hemodynamic support.

On the third day of ICU admission, her oxygen saturation suddenly dropped, requiring an increase in her fraction of inspired oxygen (FiO2) requirement to 100%. A large amount of fresh blood was noted to be coming from the endotracheal tube. Hemoglobin decreased from 9.6 to 7.5g/dl. Platelet count was 46,000 per µL, with an international normalized ratio (INR) of 1.3 and a partial thromboplastin time (PTT) of 38 seconds. Subsequently, she had a pulseless electrical activity cardiac arrest. After three cycles of cardiopulmonary resuscitation, she had a return of spontaneous circulation. An urgent bronchoscopy revealed profuse bleeding from both lungs. Uncontrolled bleeding with unstable hemodynamic status prompted the administration of endobronchial rFVIIa. A dose of 50mcg/Kg was dissolved in 50 ml of normal saline and administered via bronchoscopy into the right and left main bronchus simultaneously. Bleeding rapidly ceased within two hours, with improvement in oxygenation and hemodynamics. She was transfused two units of packed red blood cells, which increased the hemoglobin from 7.5 to 11.5 g/dL. Within twelve hours, oxygenation continued to improve, and the need for norepinephrine decreased. Over the next seven days, she was weaned off vasopressors and the ventilator, with an eventual transfer out of the ICU. 

Case 3

A 61-year-old woman with a known history of systemic lupus erythematosus (SLE) and pulmonary hypertension with suspicion for veno-occlusive disease initially presented to an outside hospital with worsening dyspnea and cough productive of green sputum. She was diagnosed with pneumonia and started on appropriate antibiotics. Her respiratory status deteriorated quickly, requiring mechanical ventilation for four days, after which she was extubated and transferred to our facility. Within one day at our institution, she again developed worsening hypoxemia and required endotracheal intubation and mechanical ventilation. Bronchoscopy and bronchoalveolar lavage were performed, which revealed DAH. She was treated with intravenous methylprednisolone 250 mg every six hours. She then developed acute renal failure requiring renal replacement therapy and plasmapheresis. Despite six cycles of plasmapheresis and high dose steroids, she continued to have DAH with worsening hypoxemia. Repeat bronchoscopy revealed gross pulmonary hemorrhage consistent with worsening DAH. It was decided to proceed with endobronchial Factor VIIa. A solution was made using 50mcg/kg of rFVIIa, which was diluted in 50mL of sterile normal saline. It was then placed into a 60cc syringe. The airways were cleaned with normal saline, and 2 ml of the rFVIIa solution was applied topically via bronchoscopy into each lung segment on the right and the left lung. The next day she had a repeat bronchoscopy, which showed resolution of DAH. Despite achieving endobronchial hemostasis, the patient continued to deteriorate and progressed to multi-organ failure, at which point the family decided to place her on comfort measures and eventually withdrew care. 

Case 4

A 22-year-old woman with a history of stage IV pulmonary sarcoidosis as well as neurosarcoidosis presented to our institution with worsening productive cough, shortness of breath, and hypoxia for two weeks duration. The initial chest radiograph showed interstitial markings and cystic changes consistent with previous films. CT angiography of the chest did not show a pulmonary embolism but did reveal peripheral honeycombing with scattered ground-glass opacities. She was started on treatment for pneumonia. Over the next twenty-four hours, she became increasingly more tachycardic and hypoxic and went into cardiac arrest with pulseless electrical activity. She was emergently intubated and resuscitated. A bronchoscopy was performed, which showed cloudy thin secretions. Given her refractory hypoxemia on maximum ventilator support, she was switched to a high-frequency oscillatory ventilation mode, which did not improve her hypoxemia. She was eventually placed on venovenous extracorporeal membrane oxygenation (ECMO). A repeat bronchoscopy showed scant bloody secretions, and sequential bronchoalveolar lavages were consistent with DAH. A solution was made using 50 mcg/kg of rFVIIa diluted in 50mL of sterile normal saline. It was then placed into a 60cc syringe. The airways were cleaned with normal saline, and 2 ml of the rFVIIa solution was applied topically via bronchoscopy into each lung segment on the right and the left lung. The patient had a repeat bronchoscopy the following day, which showed resolution of the DAH; however, multi-organ failure progressed, requiring maximum doses of three vasopressors. After one week on ECMO, the patient’s family decided to withdraw care. 

## Discussion

Diffuse alveolar hemorrhage (DAH) is characterized by hemoptysis, anemia, diffuse alveolar infiltrates, and acute respiratory failure [7]. Bleeding due to DAH originates in the pulmonary microvasculature, which includes the alveolar capillaries, arterioles, and venules [7]. The pathogenesis of DAH typically stems from capillaritis occurring in the setting of autoimmune syndromes, such as systemic lupus erythematosus (SLE) and anti-neutrophil cytoplasmic associated (ANCA) vasculitis, however, can also occur as a result of coagulopathy, stem cell transplantation, or exposure to drugs and toxins [7,8]. DAH can be categorized into three subtypes: acute macroscopic bleeding, chronic microscopic alveolar bleeding, and blast lung injury. Of the mentioned subtypes, acute macroscopic bleeding is the most aggressive and fatal; therefore, prompt recognition and initiation of treatment are crucial for patient survival [8]. 

DAH has a high in-hospital mortality rate, ranging from 20-100% [9]. Despite its name, about one-third of patients do not present with overt hemoptysis; thus, diagnosis requires a high index of suspicion [7]. Symptoms may include rapidly progressive dyspnea, cough, and in some cases, hemoptysis. A chest radiograph will typically depict diffuse bilateral infiltrates, which may resemble other disease entities, such as acute respiratory distress syndrome (ARDS) and transfusion-related acute lung injury (TRALI). Ultimately, the final diagnosis requires bronchoscopy with serial broncho-alveolar lavages progressively becoming more hemorrhagic [8].

Few randomized trials have been performed regarding the management of life-threatening DAH; therefore, treatment remains controversial. The underlying principle lies in treating the underlying etiology of DAH, such as reversing a coagulopathy in the case of a coagulation cascade defect. Treatment focuses on supportive measures such as mechanical ventilation with high positive end-expiratory pressures with the goal of providing tamponade for bleeding, as well as blood transfusions and fluid resuscitation [8]. In cases of autoimmune associated DAH, pulse doses of steroids, and immunosuppressive therapies, such as cyclophosphamide, remain the gold standard [7,10]. Anti-fibrinolytic agents, such as tranexamic acid and aminocaproic acid, have also been implemented as rescue modalities for refractory cases of DAH, and have been administered via intravenous, intrapulmonary, and nebulized routes. More recently, the use of intrapulmonary recombinant activated factor VII (rFVIIa) has been popularized, which was the basis for our treatment plans in the aforementioned cases. 

Recombinant activated factor VII was developed in the 1980s for the treatment of hemophilia and has been commercially available since 1995 [11]. In the United States, it is currently approved as a hemostatic agent for congenital hemophilia with antibody inhibitors to factors VIII or IX, acquired hemophilia, and congenital factor VII deficiency. In Europe, rFVIIa is additionally approved for the treatment of Glanzmann’s thrombasthenia [8,12]. In recent years the use of rFVIIa for life-threatening bleeding conditions outside of the approved indications has increased exponentially. Recombinant activated factor VIIa has been referred to as a “broad-spectrum hemostatic agent” [8]. At physiologic levels, factor VII plays a tissue-factor mediated role in the extrinsic coagulation pathway by activating factor X to Xa, which subsequently results in the conversion of prothrombin to thrombin, then fibrinogen to fibrin, which forms the cross-linked mesh known as the hemostatic plug. However, at the supra-physiologic doses administered with bleeding episodes, recombinant activated factor VII directly binds to activated platelets, thus by-passing the tissue-factor mediated pathway. Current use has increased more than one hundred times compared to the previous decade, with more than 90% of the uses being off-label [11]. Common uses of systemic rFVIIa include traumatic, post-operative, and post-partum hemorrhage, as well as spontaneous cerebral hemorrhage and bleeding diathesis from liver disease [11]. 

Perhaps the most feared complication of systemic administration of rFVIIa is the increased risk of thrombosis, which is mostly associated with off-label use. Some of the most commonly reported thrombotic events include arterial thrombosis, thromboembolic stroke, myocardial infarction, and venous thromboembolic disease [13]. It is postulated that local administration of rFVIIa, such as endobronchial instillation, is associated with less thrombotic risk. This is due to the hydrophilic nature of the drug, which does not allow it to penetrate the alveolar-capillary membrane, thus, ensuring a maximum effect at the local level with a lower risk of systemic effects [14]. Conversely, large doses of rFVIIa would need to be administered systemically in order to reach target sites in the alveoli in cases of DAH. 

A case series published in 2005 by Raivio et al. described the uses of systemic rFVIIa for postoperative hemorrhage. In the series, 82% of patients achieved hemostasis; however, 25% had serious thromboembolic complications [15]. 

Another important review in this matter was the Cochrane review published in 2012. The review looked at the use of systemic rFVIIa to control active bleed as well as the prophylactic use in decreasing blood loss in patients without hemophilia. In both cases, it did not show a definite decrease in mortality. It did show a decrease in blood loss in patients who received systemic rFVIIa (mean difference (MD) ‐297 mL; 95% CI ‐416 to ‐178); however, it also was associated with increased risk of thrombosis (risk ratio (RR) 1.35; 95% CI 0.82 to 2.25). The authors concluded that the use of rFVIIa should be limited to its current licensed indications and clinical trials [13].

One retrospective chart review done by Pathak et al. in 2015 looked at patients with the diagnosis of DAH who received systemic rFVIIa. Patients received 35 to 120 mcg/kg IV rFVIIa every two hours until hemostasis was achieved or treatment was judged to inadequate. Although hemostasis was achieved in 22 out of 23 patients, frequent repeated dosing of the IV formulation was required in many cases, which translated into increased cost and risk of thrombotic complications [6]. 

Despite the paucity of the data supporting its use, the use of rFVIIa has increased in recent years for other life-threatening bleeding conditions outside of the approved indications. More recently, the use of local endobronchial rFVIIa solution has gained prominence for the management of DAH. 

One question that remains unanswered is regarding the dosing and frequency that should be used when instilling endobronchial rFVIIa for the management of DAH. In the past years, a few case reports have been published regarding the successful use of endobronchial rFVIIa for DAH. It seems that lower doses, with less frequency, are needed when rFVIIa is instilled endobronchially compared to systemically [16]. The explanation seems to be related to the presence of tissue factor (TF) in inflamed alveoli. In normal lung tissue, rFVIIa binds TF to activate factor X. Factor X converts prothrombin to thrombin, which in turn activated fibrin to stop bleeding. Inflamed lungs, however, are known to contain large amounts of TF inhibitors, and therefore prevent FVIIa-TS factor formation. By instilling rFVIIa into the bronchioles, this inhibition may be overcome [16]. Data from several case series support the use of endo-bronchial rFVIIa at lower doses compared to systemic. Doses as low as 30 mcg/kg have been shown to be effective in stopping the bleed after one or doses [17-19].

The literature reports various cases of endobronchial administration of rFVIIa for refractory cases of DAH. In 2006, Heslet et al. reported six consecutive patients with DAH who were treated with endobronchial rFVIIa, which resulted in hemostasis and significantly increased PaO2:FiO2 ratios. The drug was dosed at 50 mcg/kg and was dissolved in 50 ml of normal saline [17].

It has been suggested that intra-alveolar use of rFVIIa can promote widespread alveolar fibrin deposition resulting in ARDS. The proposition is based on the known mechanisms of up-regulation of the tissue-factor-related pathway of coagulation in the alveolar compartment, contributing to lung functional impairment described in patients at risk for, or with ARDS and pneumonia [20]. However, on literature review, there has been no evidence documenting intra-alveolar thrombotic deposition after the use of systemic or intra-alveolar rFVIIa administration [20].

## Conclusions

Our case series further illustrates the effectiveness of rFVIIa for hemostasis in patients who developed DAH from several causes. The bleeding was controlled in all four cases after only one dose of rFVIIa. Most patients received a dose of 50mcg/kg diluted in 50mL of normal saline; however, one of the cases received a dose of 30mcg/kg in 50mL of normal saline with excellent results. None of the patients required repeat therapy as the bleeding was controlled after just one dose. Two of the patients in our case series had an excellent outcome with the resolution of respiratory failure, while the other two patients expired from complications not related to DAH or the use of rFVIIa. Our case series did not demonstrate any thromboembolic events or systemic adverse events after rFVIIa administration. Our data support the use of endobronchial rFVIIa use as an adjunctive treatment for DAH. Favorable results were obtained with a one-time dose of 50mcg/kg, and even a lower dose of 30mcg/kg. The use of rFVIIa may be a good therapeutic option for those patients presenting with severe, life-threatening DAH and in those failing standard therapies. 

## References

[1] Kessler C (2019). Haemostasis.com: clinical experiences in the investigational use of rFVIIa in the management of severe haemorrhage. Br J Haematol.

[2] Hoffman M, Monroe DM, Roberts HR (2019). Activated factor VII activates factors IX and X on the surface of activated platelets: thoughts on the mechanism of action of high-dose activated factor VII. Blood Coagul Fibrinolysis.

[3] Kenet G, Walden RR, Eldad A, Martinowitz U (2019). Treatment of traumatic bleeding with recombinant factor VIIa. Lancet.

[4] Simpson E, Lin Y, Stanworth S, Birchall J, Doree C, Hyde C (2019). Recombinant factor VIIa for the prevention and treatment of bleeding in patients without haemophilia. Cochrane Database Syst Rev.

[5] Hsia CC, Chin-Yee IH, McAlister VC (2019). Use of recombinant activated factor VII in patients without hemophilia: a meta-analysis of randomized control trials. Ann Surg.

[6] Pathak V, Kuhn J, Gabriel D, Barrow J, Jennette JC, Henke DC (2019). Use of activated factor VII in patients with diffuse alveolar hemorrhage: a 10 years institutional experience. Lung.

[7] Park MS (2019). Diffuse alveolar hemorrhage. Tuberc Respir Dis (Seoul).

[8] Park JA (2019). Diffuse alveolar hemorrhage and recombinant factor VIIa treatment in pediatric patients. Korean J Pediatr.

[9] De Prost N, Parrot A, Picard C (2019). Diffuse alveolar haemorrhage: factors associated with in-hospital and long-term mortality. Eur Respir J.

[10] Kusunoki M, Umegaki T, Shoji T (2019). Severe Progressive Diffuse Alveolar Hemorrhage in a Patient with Systemic Lupus Erythematosus. Case Rep Crit Care.

[11] Dutta TK, Verma SP (2019). Rational use of recombinant factor VIIa in clinical practice. Indian J Hematol Blood Transfus.

[12] Smith JE, Watts S, Spear AM, Wilson C, Kirkman E (2019). Nebulised recombinant activated factor VII (rFVIIa) does not attenuate the haemorrhagic effects of blast lung injury. J R Army Med Corps.

[13] Simpson E, Lin Y, Stanworth S, Birchall J, Doree C, Hyde C (2019). Recombinant factor VIIa for the prevention and treatment of bleeding in patients without haemophilia. Cochrane Database Syst Rev.

[14] Heslet L, Nielsen JD, Nepper-Christensen S (2019). Local pulmonary administration of factor VIIa (rFVIIa) in diffuse alveolar hemorrhage (DAH)-a review of a new treatment paradigm. Biologics.

[15] Raivio P, Suojaranta-Ylinen R, Kuitunen AH (2019). Recombinant factor VIIa in the treatment of postoperative hemorrhage after cardiac surgery. Ann Thorac Surg.

[16] Baker MS, Diab KJ, Carlos WG, Mathur P (2019). Intrapulmonary recombinant factor VII as an effective treatment for diffuse alveolar hemorrhage: a case series. J Bronchology Interv Pulmonol.

[17] Heslet L, Nielsen JD, Levi M, Sengeløv H, Johansson PI (2019). Successful pulmonary administration of activated recombinant factor VII in diffuse alveolar hemorrhage. Crit Care.

[18] Henke D, Falk RJ, Gabriel DA (2019). Successful treatment of diffuse alveolar hemorrhage with activated factor VII. Ann Intern Med.

[19] Hedner U (2019). Recombinant factor VIIa (NovoSeven®) as a hemostatic agent. Semin Hematol.

[20] Günther A, Mosavi P, Heinemann S (2019). Alveolar fibrin formation caused by enhanced procoagulant and depressed fibrinolytic capacities in severe pneumonia: comparison with the acute respiratory distress syndrome. Am J Respir Crit Care Med.

